# Hydatid Cyst in a Post‐Tuberculosis Patient: A Persistent Pulmonary Puzzle – A Case Report

**DOI:** 10.1002/ccr3.71799

**Published:** 2026-01-02

**Authors:** Abdul Rehman Shahid, Amina Asad, Janmejay Kumar Singh, Malik Muhammad Kabir, Kainat Husain, Khalil El Abdi, Fazeela Bibi, Muhammad Hassan, Bilal Aslam, Said Hamid Sadat

**Affiliations:** ^1^ Wah Medical College Wah Cantt Rawalpindi Pakistan; ^2^ Nowshera Medical College Nowshera Pakistan; ^3^ Teerthankar Mahaveer Medical College and Research Centre Moradabad India; ^4^ Wah Medical College Rawalpindi Pakistan; ^5^ Jawaharlal Nehru Medical College Aligarh India; ^6^ Faculty of Medicine and Pharmacy of Rabat, Mohammed V University Rabat Morocco; ^7^ Jinnah Medical and Dental College Karachi Pakistan; ^8^ Ayub Medical College Abbottabad Pakistan; ^9^ University of Lahore Lahore Pakistan; ^10^ Kabul University of Medical Science Abu Ali Ibn Sina Kabul Afghanistan

**Keywords:** case report, cystic echinococcosis, hemoptysis, post‐tuberculosis, pulmonary hydatid cyst

## Abstract

Pulmonary tuberculosis and hydatid cyst are two distinct pathologies with vastly different etiologies and management approaches, yet they share common risk factors and pulmonary involvement. This rare and unique case of a hydatid cyst occurring in a post‐tuberculosis patient adds to the limited literature and highlights the diagnostic and therapeutic challenges involved. A 28‐year‐old female presented with shortness of breath, productive cough, and copious hemoptysis. Clinical examination revealed decreased breath sounds on the right side. Her past medical history was significant for treating pulmonary tuberculosis, which was completed 1 year prior. Initial suspicion of TB reactivation was ruled out by negative AFB sputum analysis. Imaging revealed a well‐defined pulmonary mass, confirmed as a hydatid cyst on CT scan. The patient was managed with oxygen, IV antibiotics (cefoperazone/sulbactam), albendazole, corticosteroids, and supportive care, resulting in symptom improvement. Surgical intervention is planned for definitive management. This case is important as it highlights that in a post‐TB patient with ongoing respiratory symptoms, a hydatid cyst must be considered as a differential diagnosis, especially in regions where hydatid disease is prevalent. Diagnosing a hydatid cyst can be challenging, as both TB and hydatid cysts present with similar symptoms. Therefore, accurate diagnosis requires a multidisciplinary approach involving medical, radiological, and surgical interventions. Additionally, this case raises public awareness about the symptom overlap between hydatid cyst and TB, which can aid in early detection.

## Key Clinical Message

In post‐tuberculosis patients from endemic regions, new respiratory symptoms do not always indicate reactivation. Clinicians must consider differential diagnoses like hydatid cysts, which can mimic tuberculosis. A broad diagnostic approach, supported by advanced imaging, is crucial to avoid misdiagnosis and ensure proper management.

## 1 Introduction

In developing countries where TB and parasitic infections such as hydatid cysts coexist, they contribute significantly to morbidity and mortality. Pulmonary TB is a chronic granulomatous infection caused by *Mycobacterium tuberculosis* . It remains a major health concern in countries like Pakistan, where poor sanitation, delayed diagnosis, and inadequate follow‐up pose ongoing challenges, even with the widespread implementation of the DOTS program [1]. Hydatid disease, also known as cystic echinococcosis, is a zoonotic parasitic infection caused by the larval stage of *Echinococcus granulosus*, transmitted primarily through ingestion of eggs excreted by infected dogs.

Both pulmonary TB and hydatid cysts primarily affect the lungs and can mimic each other in clinical presentation. Shared symptoms such as chronic cough, hemoptysis, chest pain, and dyspnea often lead to misdiagnosis or delayed diagnosis, especially in post‐treatment TB patients who continue to experience respiratory symptoms [2]. Although co‐infections involving TB and hydatid disease have been reported, most involve concurrent detection at the time of initial presentation, typically in patients with compromised immunity or heavy exposure in endemic zones [3]. In contrast, sequential development of pulmonary hydatid disease following a completed and clinically resolved TB course remains extremely rare, with very few such cases documented in the global literature [4]. Structural lung damage resulting from healed TB—including fibrosis, bronchiectasis, and cavitation—may predispose patients to secondary infections, including parasitic and fungal etiologies [5]. In such post‐TB lungs, the emergence of new cystic lesions may be misinterpreted as TB reactivation, fungal ball formation, or even malignancy, particularly when conventional investigations such as sputum AFB and serological testing yield inconclusive results. In these situations, imaging studies, especially contrast‐enhanced CT, become indispensable for diagnosis [6].

This case describes a young, immunocompetent female with a history of treated pulmonary TB who presented with hemoptysis and a right‐sided pulmonary mass. Despite negative AFB results, fungal elements were identified on KOH preparation, and CT confirmed a hydatid cyst. This report illustrates the diagnostic challenge of distinguishing post‐TB sequelae from new infections and highlights the rare sequential occurrence of hydatid disease after TB. It also raises awareness about possible polymicrobial colonization in structurally damaged lungs and emphasizes the need for broad differential diagnosis and imaging in post‐TB patients with recurrent symptoms.

## 2 Case Presentation

### 2.1 Case History and Examination

A 28‐year‐old female permanent resident of Peshawar presented to the Emergency Department in February 2025 with complaints of shortness of breath on moderate exertion, cough, and copious hemoptysis for the past 3–4 days. The cough was initially dry but later became productive with fresh blood each time she coughed. She also reported chest pain that worsened when lying flat. No relevant family history, psycho‐social details, or genetic information were noted. Her past medical history was significant for pulmonary tuberculosis diagnosed in 2022, for which she completed a full course of treatment in 2023.

On admission, her vital signs were notable for oxygen saturation of 94%, respiratory rate of 34 breaths per min, pulse rate of 86 beats per min, temperature of 99°F, and blood pressure of 125/80 mmHg. Physical examination revealed that she was alert and oriented to time, place, person, and purpose, with no cyanosis. Breath sounds were decreased on the right side of the chest.

### 2.2 Investigation and Differential Diagnosis

Initial management included oxygen therapy at 4 L per min via face mask and a stat dose of intravenous dexamethasone 0.2 mg/mL. After stabilization, baseline laboratory investigations were performed, revealing a total leukocyte count of 7.68 × 10^3^/μL, hemoglobin of 12.2 g/dL, eosinophils at 2%, and neutrophils at 70%. Liver and renal function tests, serum electrolytes, coagulation profile, and urine examination were within normal limits. Arterial blood gases showed no abnormalities. Sputum was sent for acid‐fast bacilli (AFB) to evaluate for possible reactivation of tuberculosis; the results were negative with no growth of acid‐fast bacilli. However, fungal screening of the sputum using KOH preparation revealed yeast cells.

**FIGURE 1:**
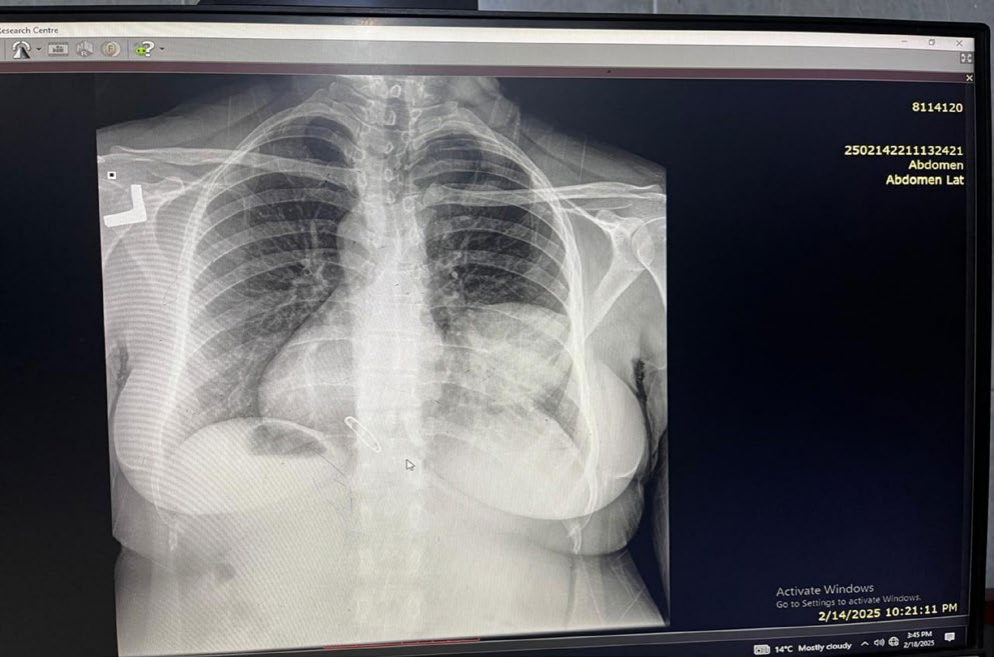
Chest X‐ray (AP View): A well‐defined mass on the right side of the chest.

**FIGURE 2:**
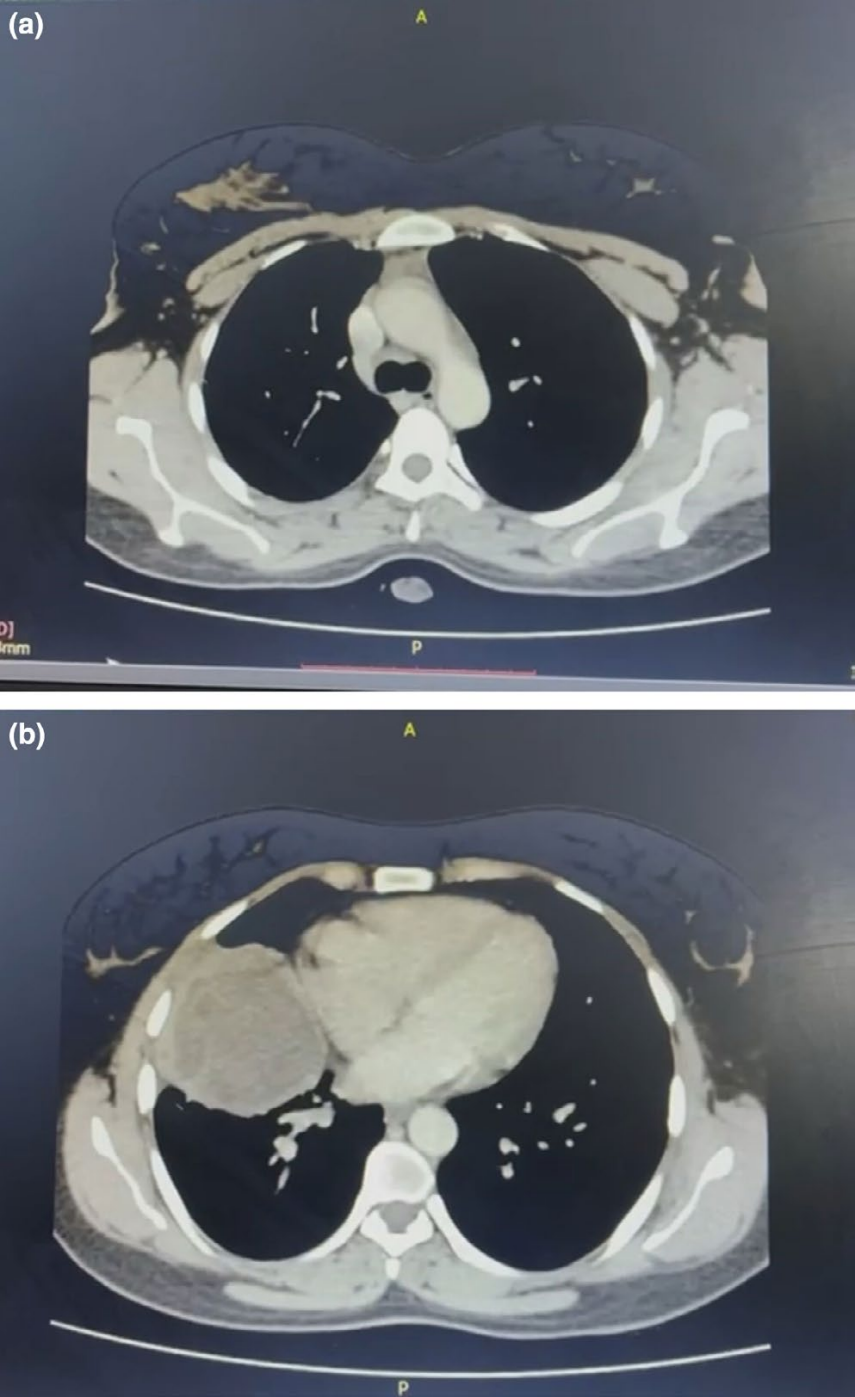
(a) CT scan of the thorax: Axial view showing a middle lobe lesion with peripheral enhancement and a central membrane, suggestive of a hydatid cyst. (b) CT scan of the thorax: Axial view showing a middle lobe lesion with peripheral enhancement and a central membrane, suggestive of a hydatid cyst.

Radiological investigations included a chest X‐ray that showed a well‐defined mass on the right side of the chest, as illustrated in Figure 1. A subsequent chest CT scan was consistent with the presence of a hydatid cyst, as shown in Figure 2a,b. The main challenge was distinguishing between TB reactivation and other causes of hemoptysis and pulmonary mass, particularly as sputum AFB was negative. Identification of fungal elements and confirmation of hydatid cysts required additional testing. Based on these findings, the working diagnosis was a pulmonary hydatid cyst, with differential diagnoses including reactivation of pulmonary tuberculosis, fungal infection, and pulmonary neoplasm.

### 2.3 Treatment

The patient was started on intravenous cefoperazone‐sulbactam 2.5 g twice daily, intravenous ketorolac three times daily for pain control, and oral albendazole 200 mg for antiparasitic treatment. Oxygen therapy was continued. Initial treatment focused on stabilizing respiratory function with oxygen and steroids. Following sputum fungal identification and imaging, albendazole was started for the hydatid cyst, alongside antibiotics to treat secondary infection. Surgical intervention is planned based on clinical response and imaging findings.

### 2.4 Follow‐Up and Outcome

The patient's shortness of breath and frequency of hemoptysis improved with medical management. Repeat laboratory tests on 21st February 2025 showed an increase in total leukocyte count to 10.2 × 10^3^/μL and eosinophils to 3%. The current treatment was continued, and she was planned for surgical and interventional radiology consultation for further management. Throughout her hospital stay, the patient tolerated all medications well, with no adverse or unexpected events reported. She demonstrated clinical improvement, and adherence to the therapeutic regimen was good.

## 3 Discussion

This case is unique due to its rare sequential presentation of a pulmonary hydatid cyst following fully treated tuberculosis, a combination infrequently reported in literature where most co‐infections occur concurrently during active TB [7, 8]. The patient's symptoms—including hemoptysis, chest pain, and dyspnea—closely resembled TB reactivation, creating a diagnostic challenge. However, the acute onset of copious hemoptysis over just 3 to 4 days was a key feature arguing against this diagnosis, as TB reactivation typically presents with a more subacute or chronic history. This atypical presentation, especially as sputum AFB was negative and imaging lacked typical TB features, underscored the need for an alternative diagnosis. CT imaging was crucial in identifying a well‐defined cystic lesion suggestive of hydatid disease, highlighting the value of advanced imaging in differentiating post‐TB sequelae from alternative pathologies [9, 10]. While the negative acid‐fast bacilli (AFB) sputum analysis was crucial in steering the initial diagnosis away from TB reactivation, it is important to acknowledge the limitations of microscopy [11]. The gold standard for TB diagnosis remains mycobacterial culture, although its long turnaround time of 2–8 weeks can delay treatment [12]. Modern molecular methods, such as the WHO‐endorsed Xpert MTB/RIF assay (a cartridge‐based nucleic acid amplification test or CBNAAT), offer a significant advantage [11]. These tests provide rapid and sensitive detection of *Mycobacterium tuberculosis* DNA and can identify rifampicin resistance within hours, overcoming the limitations of both smear microscopy and slow culture growth [13].

A notable complicating factor was the detection of yeast forms on KOH preparation, raising the possibility of secondary fungal colonization in a structurally damaged lung. Although no overt fungal infection was diagnosed, the coexistence of post‐TB changes, hydatid cyst, and possible fungal elements remains an exceptional triad in clinical literature [14, 15]. Additionally, the demographic context adds significance–this was a 28‐year‐old immunocompetent female, unlike the typical pediatric or elderly cases with underlying risk factors, emphasizing that even healthy individuals may be at risk in endemic regions like Peshawar, Pakistan [16].

This case underscores both the strength of a multidisciplinary diagnostic approach and the limitation of relying solely on conventional TB workups. It advocates for broader differentials in post‐TB patients and demonstrates how parasitic and fungal pathologies may emerge in previously infected lungs. Early imaging, comprehensive microbiological testing, and clinical vigilance are vital for timely and accurate diagnosis [9, 17].

Pulmonary hydatid disease and tuberculosis are both endemic in South Asia and often present with overlapping clinical and radiological features. However, their sequential occurrence is rarely documented. Echinococcus granulosus, the causative agent of hydatid disease, can infect structurally compromised lungs, particularly in post‐TB settings where fibrosis and cavitation promote secondary colonization [18].

While previous literature has documented cases of concurrent TB and hydatid disease, such as those by Laldayal et al. and Jalayeri et al., these involved simultaneous diagnoses [19, 20]. In contrast, our case represents a sequential presentation in an immunocompetent young adult–an uncommon demographic for such complications [21]. This highlights that even healthy individuals may develop opportunistic infections in the context of post‐TB pulmonary damage.

The presence of fungal elements in sputum microscopy, although not indicative of active infection, raises the possibility of polymicrobial colonization of cysts or cavities in previously diseased lungs [21, 22]. Radiologically, while the chest X‐ray suggested a nonspecific mass, contrast‐enhanced CT helped identify the lesion as a hydatid cyst, consistent with diagnostic features described by Pedrosa et al. [23].

Pulmonary tuberculosis (TB) and hydatid disease are endemic in regions with poor sanitation and close animal contact. Though caused by distinct pathogens, they share overlapping clinical presentations such as chronic cough, hemoptysis, and chest pain, which can complicate diagnosis in post‐TB patients. In those with structural lung damage from prior TB, hydatid cysts may develop and be misinterpreted as TB reactivation or other pathologies unless imaging and microbiological testing are pursued [24, 25].

Reports by Laldayal et al. and Jalayeri et al. have emphasized the diagnostic confusion in cases of co‐infection, where imaging and bronchoalveolar lavage were essential for identifying multiple pathogens [26, 27]. Similar findings in paediatric and immunocompromised patients highlight the importance of a multidisciplinary approach in persistent or atypical cases [28].

CT imaging is crucial for differentiating hydatid cysts from post‐TB sequelae, with features like fluid‐filled, well‐defined lesions often pointing to echinococcosis [29]. Though serology and eosinophilia may aid in diagnosis, they are not definitive [30]. In our patient, new‐onset symptoms post‐TB, imaging suggestive of hydatid disease, and fungal elements in sputum supported a multifactorial etiology [28, 31].

Treatment requires tailored management. Albendazole remains first‐line for uncomplicated hydatid disease, while surgical intervention is considered for large or symptomatic cysts [32]. Co‐infections demand individualized antimicrobial regimens and a coordinated, multidisciplinary approach [33, 34]. This case also underscores the need for expanded diagnostic algorithms and preventive strategies in TB‐endemic areas to account for parasitic and fungal diseases [12].

## 4 Conclusion

This case highlights that in post‐tubercular patients with persistent pulmonary symptoms, a diagnostic dilemma may arise, especially in regions where parasitic infections such as hydatid cyst are endemic. Other documented cases have shown the coexistence of TB and hydatid cyst; however, this case is unusual and unique as it demonstrates a delayed parasitic infection by *Echinococcus*, which mimicked TB reactivation. This case also emphasizes the need to consider a broad differential diagnosis when a patient presents with hemoptysis or a pulmonary mass. Imaging, especially contrast‐enhanced CT (CECT), should be used judiciously to differentiate between hydatid cyst and TB. Furthermore, additional investigations should be pursued if fungal elements are detected in the sputum. This case further emphasizes the importance of a multidisciplinary approach, where clinical history, imaging, and microbiological screening are all essential for accurate diagnosis. It also advocates for increased public awareness, especially in endemic areas, where post‐TB patients should be informed about the risk of parasitic infections that can coexist with or occur even after successful TB treatment. To our knowledge, this is among the first reported cases of post‐tuberculosis pulmonary hydatid cyst in an immunocompetent adult in South Asia, making it a valuable contribution to the growing body of literature on complex infectious pulmonary pathology.

## Author Contributions

**Abdul Rehman Shahid:** conceptualization, data curation, writing – original draft. **Amina Asad:** formal analysis, investigation, validation. **Janmejay Kumar Singh:** investigation, methodology, project administration. **Malik Muhammad Kabir:** resources, software, visualization. **Kainat Husain:** formal analysis, project administration, writing – review and editing. **Khalil El Abdi:** conceptualization, project administration, resources, validation, writing – original draft, writing – review and editing. **Fazeela Bibi:** supervision, validation, writing – review and editing. **Muhammad Hassan:** project administration, supervision. **Bilal Aslam:** formal analysis, investigation, methodology. **Said Hamid Sadat:** writing – review and editing.

## Funding

The authors have nothing to report.

## Ethics Statement

The authors have nothing to report.

## Consent

Written informed consent was obtained from the patient to publish this report in accordance with the journal's patient consent policy.

## Conflicts of Interest

The authors declare no conflicts of interest.

## Data Availability

The data was taken from a patient who presented to our hospital, all data and references are publicly available on databases such as Pub‐med and Google Scholar.
